# Methods and approaches for enhancing communication with people with moderate-to-severe dementia that can facilitate their inclusion in research and service evaluation: Findings from the IDEAL programme

**DOI:** 10.1177/14713012211069449

**Published:** 2022-02-13

**Authors:** Rachel Collins, Anna Hunt, Catherine Quinn, Anthony Martyr, Claire Pentecost, Linda Clare

**Affiliations:** College of Medicine and Health, 3286 University of Exeter, Exeter, UK; The Centre for Applied Dementia Studies, 1905 University of Bradford, Bradford, UK; Wolfson Centre for Applied Health Research, Bradford, UK; College of Medicine and Health, 3286 University of Exeter, Exeter, UK; College of Medicine and Health, 3286 University of Exeter, Exeter, UK; 3286 NIHR Applied Research Collaboration South-West Peninsula, Exeter, UK

**Keywords:** qualitative research, framework analysis, living well, quality of life, interlocutor

## Abstract

**Objectives:**

Dementia can affect language processing and production, making communication more difficult. This creates challenges for including the person’s perspective in research and service evaluation. This study aims to identify methods, tools and approaches that could facilitate meaningful communication with people with moderate-to-severe dementia and support the inclusion of their perspectives.

**Methods:**

This qualitative study was conducted as part of the IDEAL programme and involved in-depth, semi-structured interviews with 17 dementia research and/or care professionals with expertise in communication. Transcripts were analysed using framework analysis.

**Findings:**

Three main themes each with sub-themes were identified: (1) Awareness, knowledge and experience; (2) Communication approach and (3) Personalization. A person-centred orientation based on getting to know the participant and developing a bi-directional exchange formed the fundamental context for effective communication. Building on this foundation, an approach using pictures, photographs or objects that are meaningful to the person and appropriate for that person’s preferences and ability could help to facilitate conversations. The findings were integrated into a diagram illustrating how the topics covered by the themes interrelate to facilitate communication.

**Conclusions:**

Useful skills and approaches were identified to help researchers engage and work with people with moderate-to-severe dementia and ensure their perspective is included. These covered getting to know the participant, using a variety of tangible tools and interactional techniques and considering the environment and context of the conversation.

## Introduction

Fifty million people live with dementia worldwide with the number estimated to rise to 152 million by 2050 (Alzheimer’s Disease International, 2019). Understanding experiences and assessing the ability to ‘live well’ for those diagnosed with dementia are high priorities for research, policy and practice (Department of Health, 2009; Institute of Medicine, 2012). To develop effective person-centred care, services, interventions and support, people with moderate-to-severe dementia need to be included in research and their opinions and perspectives sought. Service provision can then be developed from the perspective and experience of service users (Beresford, 2013; Cheston et al., 2000; Gove et al., 2018; Murphy et al., 2015; Smebye et al., 2012). A limited number of studies have directly elicited the perspectives, experiences and opinions of people with moderate-to-severe dementia (Cahill & Diaz-Ponce, 2011; Clare et al., 2008a; Hughes, 2013; Williamson, 2010), but there is still a paucity of research and there is a need for a better understanding of how to elicit their viewpoints.

To address specific care needs, communication between researchers or health care professionals and people with dementia becomes increasingly important as dementia severity increases (Gove et al., 2018; Smebye et al., 2012). With advancing dementia, the ability to communicate can decrease due to cognitive and linguistic decline, leading to potential challenges in being understood. Different challenges may be experienced depending on dementia subtype and progression (Boschi et al., 2017). For example, difficulties in word-finding and verbal expression can lead to frustration and distress due to the inability to verbally express or convey thoughts and feelings (Downs & Bowers, 2014). Communication difficulties can increase the risk of individual needs not being met and so may also directly impact on living well. Limited communication may exacerbate depression, anxiety and loneliness (Downs & Collins, 2015) and contribute to physical and cognitive decline (Skov Uldall et al., 2012). Where feelings of frustration are expressed and behaviours arising from unmet care needs are displayed, others may perceive these as challenging behaviour (Duxbury et al., 2013; Hughes, 2013). Although expression by verbal means may be more limited, non-verbal communication can convey feelings, emotions and preferences (Ellis & Astell, 2017; Quinn et al., 2014). Non-verbal communication is often preserved in people with moderate-to-severe dementia, while the desire and ability to communicate opinions and answer questions is maintained (Astell & Ellis, 2006; Brandão et al., 2014; Clare et al., 2008a; Ellis & Astell, 2004; Moore & Hollett, 2003).

As the number of people with moderate-to-severe dementia increases, researchers, family members and healthcare workers need methods and approaches to facilitate meaningful communication with this population to enable their involvement in research and service development. Given the relative preservation of non-verbal communication skills, this could potentially involve non-verbal techniques augmenting or enriching verbal communication or a combination of both. Although there are effective communication methodologies for those with less severe dementia and for stimulating general social engagement (Alsawy et al., 2017; Egan et al., 2010; Kindell et al., 2017), research aimed at communicating with people with moderate-to-severe dementia, especially to gauge health or well-being status and care preferences, is more limited (Williamson, 2010).

Strategies and methods to facilitate general communication in people with dementia have been identified and reviewed (Egan et al., 2010); appropriate methods include using short sentences and eliminating distractions (Alsawy et al., 2017), activity-based approaches and programmes that train caregivers (Egan et al., 2010; Eggenberger et al., 2013; Swan et al., 2018). Several tangible tools and external memory aids (Augmentative and Alternative Communication), are also available. These include memory and picture books, and technologically based tools such as the Computer Interactive Reminiscence and Conversation Aid (Alm et al., 2004; Arnott & Alm, 2016; Astell et al., 2010; May et al., 2019). While most tools have been utilized to stimulate general conversations or improve social interaction, Talking Mats (Murphy et al., 2007a, 2010) provide some opportunity to elicit opinions and preferences (Murphy et al., 2007a, 2010; Williamson, 2010) and facilitate decision-making (Murphy & Oliver, 2013; Murphy et al., 2010) but they may not be effective for everybody with moderate-to-severe dementia (Williamson, 2010).

In the Improving the experience of Dementia and Enhancing Active Life (IDEAL) programme the perspectives of people with mild-to-moderate dementia were elicited (Clare et al., 2014). The follow-on IDEAL-2 study (Silarova et al., 2018) aimed to build on this by exploring ways of including the perspectives of people with moderate-to-severe dementia. Here, we aim to identify methods, tools and approaches currently known or used in professional practice that could be used in future research to elicit responses of people with moderate-to-severe dementia.

## Methods

### Design

This exploratory study used semi-structured in-depth interviews. Data were analysed using framework analysis (Gale et al., 2013)

### Ethical approval

Ethical approval to conduct this study in England and Wales was obtained from the Wales Research Ethics Committee 5 - Bangor (18/WA/0111). To maintain confidentiality, participants’ details were anonymized and personal information redacted from transcripts.

### Setting and sampling

A purposive sampling approach was adopted to recruit experts from a range of professional backgrounds including academia and health care, who were likely to provide rich and varied information about communication strategies for people with moderate-to-severe dementia. Seventeen professionals with experience in dementia research and/or care and expertise in communication participated in this study (interviewees). Potential interviewees were identified by pragmatic sampling via professional connections and networks across academic, health and social care settings. Potential interviewees were sent an invitation letter and information sheet explaining the study and inviting them to take part. Fourteen individual interviews were undertaken and one interview was conducted jointly with three interviewees from the same institution. All interviews were conducted by a University of Exeter researcher.

Interviewees were asked open-ended questions over the telephone using a semi-structured schedule (see Supplementary Table 1). The interviewees were asked about their experience, knowledge and expertise in communicating with people with moderate-to-severe dementia. Their thoughts and experience of specific tools, resources or techniques for communication and how to facilitate communication in different settings and with different audiences were elicited. The interview schedule allowed comparisons to be made across the interviews. The interviews were conducted between April and August 2018 and were on average 53 min long, ranging from 25 and 88 min according to the response style of each interviewee. All interviewees provided written informed consent. All interviews were recorded and professionally transcribed for analysis.

### Data analysis

Transcripts of interviews were imported into NVivo 12. To allow for researchers’ interpretations of interviewees’ experiences to be transparent and the identification of both derivable and emergent themes (Ritchie & Spencer, 2002), the seven-stage framework analysis approach (Gale et al., 2013) was used. This analysis approach facilitated the identification and analysis of emerging themes from the data and cross-referencing of themes across the interviews to identify commonalities and differences across interviewee responses. Following transcription, two researchers not involved in data collection (RC, AH) undertook six stages: familiarization, coding, developing a working analytical framework, indexing, charting and mapping and interpretation (Gale et al., 2013). Familiarization was achieved through repeated reading of the transcripts which provided a broad overview of interviewees’ responses. Open coding of four randomly selected interviews in NVivo 12 was undertaken (RC), two of which were independently coded by a second researcher (AH). The codes were discussed and final codes and initial categories were agreed. A working analytical framework was developed, comprising 18 categories. This framework was used to index the remaining interviews. The analytical framework was revised by making comparisons both within and between cases and re-classifying any codes as required, and the final nine themes were developed, discussed and agreed (RC, AH) (see Supplementary Table 2). Comparison of two of the interviews demonstrated an inter-rater reliability of 70%, which can be defined as moderate-to-substantial (Landis & Koch, 1977; McHugh, 2012).

To ensure trustworthiness, initial coding and theme development was discussed with the wider research team and any disagreements resolved.

### Mapping and interpretation

All instances of the themes and sub-themes were reviewed again and mapped across each of the interviews. A matrix was created to show which themes and sub-themes were captured in each interview. Once the themes and sub-themes had been established, a coherent diagram was developed going beyond listing the themes by illustrating how they relate to each other and how they capture the process of having the conversation. This places the themes in a wider context that could be used as a resource by researchers or practitioners wanting to hold conversations with people with moderate-to-severe dementia to elicit their views and opinions. To start the process of developing the diagram, each interlocutor was considered in turn. The researchers reviewed and reflected on every theme and sub-theme to determine first whether and how it influenced each individual involved in communicating, second whether it influenced both interlocutors simultaneously and third what overall impact it had on the communication process. Concurrently, the researchers examined whether the themes and sub-themes followed a sequential order in progressing through the communication process or reflected feedback loops. Based on these processes, visualization was created to integrate the themes and sub-themes, placing them in context. Each stage of the development of the diagram was discussed with the IDEAL-2 team, modified and agreed.

## Findings

### Participants

Table 1 summarizes the characteristics of the 17 (12 females) interviewees. Most of the interviewees were academics or clinicians. Fourteen of the 17 interviewees gave the number of years they had worked with people with dementia. This ranged from 1 to 43 years.

**Table 1.** Characteristics of the professional interviewees.

Interview no. (interviewee no.)	Gender	Profession	Approximate no. Years of experience
1 (1)	F	Academic (retired)	Not stated
2 (2)	M	Dementia researcher (non-academic)	Not stated
3 (3)	F	Academic	40+
4 (4)	F	Academic	17
5 (5)	F	Academic	20+
6 (6)	F	Adult social care inspector	34
7 (7)	F	Academic/writer	22
8 (8)	M	Occupational therapist	18
9 (9)	F	Consultant clinical psychologist	30
10 (10)	F	Clinical academic (clinical psychologist)	20+
10 (11)	F	Clinical academic	6+
10 (12)	F	Clinical academic	1+
11 (13)	M	Academic/writer	26
12 (14)	M	Academic	43
13 (15)	F	Care for older people charity manager	>4
14 (16)	M	Academic	Not stated
15 (17)	F	Clinical academic (speech and language therapist)	20+

### Thematic outcomes

Using an inductive approach three main themes were identified: (1) Awareness, knowledge and experience; (2) Communication approach and (3) Personalization. The main themes and sub-themes are seen in Table 2. Themes were consistent across the different professions and sexes of the interviewees.

**Table 2.** Three main themes with corresponding sub-themes.

Theme	Sub-theme
1. Awareness, knowledge and experience	1a Cultural or social contexts and challenges
	1b Transferable approaches
	1c Creative thinking and approaches
2. Communication approach	2a Humanity and compassion
	2b Tangible techniques
	2c Interactional techniques
3. Personalization	3a Environment and setting
	3b Getting to know each other
	3c Person-centred approach

### Theme 1: Awareness, knowledge and experience

This theme encompasses several factors that were considered important considerations for anyone wishing to engage with a person with moderate-to-severe dementia, before any attempt at communication being made. The interviewees emphasized that the process of communicating with people with moderate-to-severe dementia does not begin at the point of interaction and cannot be achieved by using a communication tool or tools alone.

### Cultural or social contexts and challenges

The challenges of working in the field of moderate-to-severe dementia care were widely acknowledged by participants, as was the need for experiential training for researchers.

“…as a novice you tend to rely on tools a lot more than you tend to rely on your own ability and you haven’t always at that stage got the confidence and rely on your own things and let that interview develop”. (Interviewee 11)

In addition, many identified social and cultural factors, such as societal stigma and internally held assumptions, which may unconsciously impact communication with people with moderate-to-severe dementia. The importance of acknowledging and reflecting on these factors as a way of improving communicative abilities was stressed.

“We often make too many assumptions about how to approach [people with moderate-to-severe dementia] and what to talk to them about. You can induce distress by making those assumptions”. (Interviewee 3)

The combination of dealing with assumptions about people with dementia and the need for knowledge and training was also highlighted.

“I would always be very wary about going to tools and techniques before you have learnt how to be with somebody with dementia. It is about being comfortable with people with more advanced dementia and so I would recommend this to anybody who is researching in this field”. (Interviewee 10)

### Transferable approaches

There is a potential utility of drawing parallels between people with dementia and other populations who may also experience communication difficulties. Many interviewees identified potential insights or techniques that have been used in fields such as stroke or learning difficulties where communication techniques are perhaps better understood or more developed than in the field of dementia.

“People are unaware of the communicative capabilities of people with advanced dementia… in people with autism or young infants, if they are engaging in repetitive behaviors, they are always credited with some meaning, but in advanced dementia they are not. They are often thought of as meaningless, random or problematic”. (Interviewee 4)

Despite many interviewees highlighting the potential benefit of drawing on lessons learnt from working with other populations, challenges were identified.

“There can be a little bit of resistance … between disability groups about using mediums from other groups and to some extent that’s understandable”. (Interviewee 2)

### Flexibility and adaptability

The importance of flexibility, adaptability and creativity when communicating with people with moderate-to-severe dementia was consistently highlighted. It was widely acknowledged that a uniform approach to communication is ineffective.

“I think it is about having a range of options … like having a kind of tool bag and you need the right elements to meet the person’s needs at the time … if that doesn’t seem helpful today then what else might I try that will be helpful to improve communication with the person?” (Interviewee 9)

This may require the interviewer to think more creatively using visual cues to stimulate a conversation, for example, a colourful tie might help to stimulate a conversation.

“If I am doing interviews I will often wear a big piece of jewelry or a big brooch...it can start a conversation and because it is near your face, helps people to achieve eye contact with you”. (Interviewee 10)

It was emphasized that the person trying to communicate with the person with moderate-to-severe dementia should be open to trying new methods and not be disheartened if a given method does not work. This is where experience can help to expand potential tools and methods by giving the interviewer the confidence to use them.

“It’s just adapting to what that person can understand and it’s just an exercise and trying different modes of communication until you find the right one”. (Interviewee 8)

### Theme 2: Communication approach

This theme incorporates techniques, practises and practical strategies that interviewees highlighted from their own experience as being effective for initiating and stimulating a conversation with people with moderate-to-severe dementia.

### Humanity and compassion

The approach and demeanour of the person initiating the conversation was viewed as a vital component for communication in itself.

“I think the main tool that you have is…your humanness and your ability to interact and observe and mirror and pace”. (Interviewee 10)

An empathetic, understanding and caring approach was felt to generate confidence and build trust, allowing the person with dementia to feel at ease, thus supporting the initial development of any conversation.

“[An interviewer] has the capacity to let know, in a variety of means, that [he/she] is available, [he/she] is truly present and available and interested in whatever they want to offer”. (Interviewee 7)

### Tangible techniques

Several specific tools and techniques were identified as useful for initiating and engaging in conversations, including using newspapers and magazines, and more activity-based methods such as poetry, art, music and drama, especially for reminiscence stimulation. However, pictures, picture books, photographs, videos, symbols and objects were consistently endorsed as being more effective for stimulating conversations and obtaining personal opinion.

“Often, objects will get more of a conversation going. So you know, personalized objects”. (Interviewee 10)

This was especially in conjunction with tools such as Talking Mats.

“[Talking Mats] cards with picture images to convey messages ... it was a meaningful way of gathering information for people with more severe cognitive impairment to whom a conversation or being asked questions was difficult without any prompts”. (Interviewee 2)

Although considered an effective tool, challenges of using Talking Mats were also identified including level of complexity, restricted and standardized options and cost and training requirements. Various challenges of using images were also highlighted, not only for the visually impaired but also for conveying more abstract concepts such as quality of life.

“One of the cards we used, possibly for feeling free to do what you want, was a kite, as in flying your kite, to try and indicate freedom, but I think one or two people said, ‘That’s a kite’. Quite justifiably”. (Interviewee 2)

Some also felt cartoons or symbols could be construed as patronizing and inappropriate for adults, though this may depend on culture, referencing the popularity of cartoons in Japan. Some felt that photographs were a valuable source for stimulating conversation, though reservation was expressed if the photograph was not personal to the person with moderate-to-severe dementia or the photograph was not congruous with their current timeframe, for example, a photograph of the person’s children as adults when the person was remembering them as children.

The potential of using technology to aid communication was also discussed. The majority had never used technology to aid communication with people with moderate-to-severe dementia but could see the potential value in iPads or other handheld devices that researchers could use to display personalized resources such as photographs to aid communication and decision-making.

### Interactional techniques

The interviewees also highlighted numerous interactional techniques that facilitated verbal communication with people with moderate-to-severe dementia, such as giving enough time, using short and simple sentences, avoiding asking direct questions and reflecting on or identifying non-verbal signs.

“If they haven’t got the words for that, I might reflect back and say ‘you are looking cross’ or ‘do you feel unhappy when that happens?’ and they maybe nod, or give me a thumbs up”. (Interviewee 9)

The importance of searching for the meaning in how the person with moderate-to-severe dementia responds, whether verbal or non-verbal, was also highlighted.

“Interviewing a lady …[she] kept going on about the snake on the door…I remember thinking she must be delirious…or having visual hallucinations…my supervisor said, ‘well is she Scottish?’… ‘in Scotland the snake on the door is the catch’, so she was actually saying ‘I want to get out of this place". (Interviewee 10)

In addition, using appropriate humour and compassion was also considered an important asset to use to build rapport.

### Environment and setting

All interviewees acknowledged the importance of considering the environment in which the communication takes place. Many recounted experiences of engaging with people with moderate-to-severe dementia in busy and/or unfamiliar environments causing distraction and/or distress to the person with moderate-to-severe dementia. In contrast, being in a familiar environment, with their belongings around them, helped to put people with dementia at ease and induce conversation.

“...the standard sensory information, thinking about the lighting, where you sit and how close you are to the person. Those kind of auditory and good practice principles we need to take into account”. (Interviewee 9)

When considering the environment, interviewees highlighted the importance of acknowledging and managing the potential influence of other people who may be present within that environment, such as family members or care staff. Both positive influences, such as affirmation and encouragement, and negative influences, such as overbearing behaviour towards the person with moderate-to-severe dementia, were identified. Interviewees emphasized any such influences should be considered and managed accordingly.

“[Spouses] some managed it very well but some … didn’t want to hear what the person with dementia was saying … wanted to sort of impose their views a bit too much”. (Interviewee 17)

### Theme 3: Personalization

Interviewees unanimously stated that the key to any conversation was maintaining an individualized approach with the person.

### Getting to know each other

Getting to know one another beforehand was considered fundamental for a successful conversation. A key phrase was ‘taking the time to get to know the person’. This could be achieved through gradual engagement in small, informal conversations and/or speaking to care staff and/or relatives. The use of observation was also emphasized, as a person’s mannerisms, behaviour patterns, likes and dislikes, hobbies and interests, personal history, even topics to avoid, could be elicited prior to starting the conversation.

“… know as much about the person that you are going to be speaking to, as possible … what their communication style is … have got particular issues … you can do a lot of preparation before you actually sit down with somebody”. (Interviewee 10)

### A person-centred approach

The importance of placing the person with moderate-to-severe dementia at the centre of any communication with the overarching aim of helping them to express their opinion, needs and wants was central to all the interviews.

“People don’t go through dementia in orderly stages, and everybody is different, the real thing is tuning into the individual”. (Interviewee 1)

The interviewees described using communication tools and methods tailored to the individual, using different approaches as necessary and working at the pace of the individual. This highlights the need for flexibility and adaptability in the communicative approach, and the potential utility of adapting or creating new methods of communication to help the individual to express themselves.

The interviewees stated that being person-centred ensured the nature and content of the conversation was not only appropriate for somebody with moderate-to-severe dementia but was also consistent with their age, sex, culture, ability and personal history.

“You can’t have a one-size-fits-all communication care plan because everybody is different … any activity needs to be bespoke to the individual. If I was communicating with someone whose first language was Urdu, they didn’t have any formal education, had dementia, lived in the middle of Manchester, I wouldn’t have the same communication approach as if it was someone living in Guildford, who was an ex-GP”. (Interviewee 3)

Each theme and sub-theme were identified in most interviews, demonstrating their stability and consistency (Table 3). This helps to illustrate the consensus of the overriding methods, tools and approaches provided by the interviewees.

**Table 3.** Matrix showing the identified themes and how they appeared in the interviews.

Interview	Themes								
	Awareness, knowledge and experience			Communication approach			Personalization		
	Cultural or social contexts and challenges	Transferable approaches	Creative thinking and approaches	Humanity and compassion	Tangible techniques	Interactional techniques	Environment and setting	Getting to know each other	Person-centred approach
1	**+**	**+**	**+**	**+**	**+**	**+**	**+**	**+**	**+**
2	**+**	**+**		**+**	**+**	**+**	**+**	**+**	**+**
3	**+**	**+**	**+**	**+**	**+**	**+**	**+**	**+**	**+**
4	**+**	**+**		**+**	**+**	**+**	**+**	**+**	**+**
5	**+**	**+**	**+**	**+**	**+**	**+**	**+**	**+**	**+**
6	**+**		**+**	**+**	**+**	**+**	**+**	**+**	**+**
7	**+**	**+**	**+**	**+**	**+**	**+**	**+**		**+**
8		**+**	**+**		**+**	**+**	**+**	**+**	**+**
9	**+**	**+**	**+**		**+**	**+**	**+**	**+**	**+**
10	**+**	**+**	**+**	**+**	**+**	**+**	**+**	**+**	**+**
11	**+**	**+**	**+**	**+**	**+**	**+**	**+**	**+**	**+**
12	**+**		**+**	**+**	**+**	**+**	**+**	**+**	**+**
13	**+**	**+**	**+**	**+**	**+**	**+**	**+**	**+**	**+**
14	**+**	**+**	**+**	**+**	**+**	**+**	**+**	**+**	**+**
15	**+**	**+**		**+**	**+**	**+**	**+**	**+**	**+**

Key: **+** = theme identified.

### Using the themes to visualize the communication process

**Figure 1.**
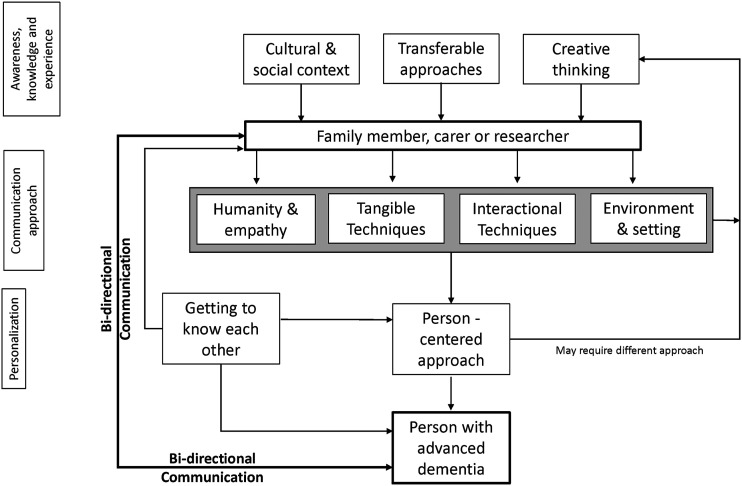
Diagram to visualize the process of communicating with people with moderate-to-severe dementia.

From the themes and sub-themes we created a diagram to visualize the interrelationships between the themes and place them in context (Figure 1). The diagram demonstrates how the issues covered in each sub-theme can influence the participants in, and the context of, the conversation. It suggests routes by which communication can be enhanced, such as taking time to get to know each other, or thinking creatively to identify ways of overcoming barriers, and shows how each element contributes to achieving a meaningful conversation.

## Discussion

This is one of the few studies to identify available tools, methods and techniques for facilitating meaningful communication with people with moderate-to-severe dementia to promote their inclusion in research and service evaluation. Three overarching themes identifying approaches to communication were elicited: (1) Awareness, knowledge and experience, (2) Communication approach and (3) Personalization. The findings indicated the fundamental importance of person-centred conversations, getting to know the participant and developing a bi-directional conversation. Building on this foundation, there was evidence for the potential utility of communication, based around personalized pictures, photographs or objects, and adapting them to the preferences and abilities of each person with moderate-to-severe dementia. The findings were integrated into a diagram illustrating how the topics covered by the themes interrelate to facilitate communication.

This study highlighted a need for awareness and understanding of potential social and cultural constraints, including negative assumptions and attitudes that can lead to the belief that people with moderate-to-severe dementia lack the ability or desire to communicate (Moore & Hollett, 2003). Previous research has shown that these beliefs can lead to people with moderate-to-severe dementia being either excluded from research or poorly supported during the research process (Alsawy et al., 2017; Alzheimer’s Disease International, 2019; Astell & Ellis, 2006; Godwin, 2014; Moore & Hollett, 2003). Awareness and management of these underlying beliefs is therefore a recommendation for anybody wishing to start working with people with moderate-or-severe dementia. As we have seen, such management, however, requires confidence, experience and skill.

For communication to be achieved tools and techniques need to be utilized that engage and support the person with moderate-to-severe dementia. Talking Mats (Murphy et al., 2007a, 2010) was considered to be an effective tool and has been shown to aid communication, participant engagement and decision-making (Arnott & Alm, 2016; Murphy et al., 2007a, 2007b, 2010). Talking Mats was not considered suitable for everybody. Some studies have suggested that with suitable adaptation, Talking Mats can be used with those with moderate-to-severe dementia to gauge opinion, although the evidence for this is limited and barriers were identified including cost and training requirements (Arnott & Alm, 2016; May et al., 2019; Murphy et al., 2007a, 2010; Williamson, 2010). Consistent with an earlier study, trying to convey complex concepts using simplified images and symbols was also acknowledged as a challenge in people with moderate-to-severe dementia (Williamson, 2010).

Other visual methods were considered powerful aids for stimulating conversations and eliciting factual information, consistent with previous research (Bourgeois et al., 2003; Brandão et al., 2014). Personalized images, for example, could be preferable due to requiring lower cognitive effort compared to generic pictures (Brandão et al., 2014; Dijkstra et al., 2006) but the choice of image and text needs careful consideration (Bate, 2012). Consistent with the current study, Small and Gutman (2002) identified short written texts as effective which may be useful when combined with pictures for reinforcement or expansion (Bate, 2012). Creative activities such as music or drama were also identified as being useful for general conversation. A diverse range of activities were suggested by interviewees; therefore, conclusions or recommendations for specific activities cannot be made. This indicates the opportunity for creative methods to improve social interaction and engagement (Livingston et al., 2014). However, these approaches may be more limited for research or service evaluation purposes where individual need and personal opinions are often sought (Godwin, 2014; Möhler et al., 2018).

Interactional techniques were considered essential when combined with more tangible methods. Non-verbal communication, such as facial expressions and body language, often allows contextualization of verbal communication (Downs & Collins, 2015). Previous research has shown that the ability to express emotions and awareness may be preserved in individuals with moderate-to-severe dementia (Karger, 2018; Quinn et al., 2014). The importance of communicating using non-verbal techniques was commonly cited including reference to palliative care. However, interpreting what is being conveyed requires skill and effort (Clare et al., 2008a, 2008b, 2014; Downs & Collins, 2015; Ellis & Astell, 2017; Quinn et al., 2014; Round et al., 2014) and links to the need for experience, skill and confidence or appropriate training for the less experienced.

Regardless of the tools and techniques selected, ensuring the conversation and approach is centred on individual need was a fundamental aspect of communication in this study. Adopting a personal approach necessitates getting to know the person prior to the conversation so that appropriate tools, methods, techniques and resources can be identified including knowing the most appropriate time and place to undertake the conversation (Clare et al., 2008b; Small & Gutman, 2002). It should also be an opportunity for the person with moderate-to-severe dementia to get to know the other interlocutor. Building a positive relationship has been shown to aid interpretation of non-verbal expressions so that the underlying meanings behind the expressions can be understood (Ward et al., 2008). Such an approach leads to a bi-directional conversation; a finding consistent with the current study and previous research (Alsawy et al., 2020).

Alongside the other social and contextual challenges identified by the interviewees, current service and research structures make the inclusion of people with moderate-to-severe dementia in research challenging. In addition to the normal pressures of researcher time, procedures and training needs, any extra burden or disruption to professional care teams should be considered (Murphy et al., 2015; Webb et al., 2020). Tensions may also occur if a perceived hierarchy arises between the interlocutors (Alsawy et al., 2020; Mann & Hung, 2019; Williams et al., 2020). As emphasized in the current findings, by getting to know each other, trust can develop between the two parties, which may help to avoid any risk of unequal power balance (Williams et al., 2020). Finally, a specific challenge in research and service evaluation is ensuring any opinions or perspectives elicited are representative (Beresford, 2007). Difficulty therefore arises when trying to move from the individual experience to a ‘collective’ voice (Williams et al., 2020). Careful management should avoid involving just actively engaged participants and instead seek to obtain a diversity of views (Beresford, 2007; Mann & Hung, 2019; Williams et al., 2020). By using the effective methods of communication identified in this study, researchers could elicit more diverse perspectives from people with moderate-to-severe dementia.

### Strengths and limitations

This study goes beyond the simple identification of potentially effective communication tools but instead demonstrates the need for a holistic approach that identifies influential and facilitating factors for both interlocutors. A limitation, consistent with other research in this area, is that the current study did not examine preferred communication methods from people with moderate-to-severe dementia themselves (Alsawy et al., 2020). However, by identifying useful methods of communicating with people with moderate-to severe dementia this study can help researchers to understand how to effectively obtain the perspectives of this hard-to-reach group in the future.

### Practice implications

Our diagram illustrates how meaningful conversation with people with moderate-to-severe dementia could be achieved by considering how the different elements interlink and could be used for developing communication strategies or identifying training needs. The tools, methods and techniques identified in this study can be used to develop effective communication strategies into a suite of tools. One example could be to develop tools that utilize pictures, photographs or objects tailored to each participant’s personal preferences and ability. Not all tools and techniques will be suitable for every individual or every time, and so a modifiable suite is recommended to allow adaptation, for example, as dementia further progresses. It is important for researchers not only to have knowledge of different tools and techniques, but also to develop the ability and confidence to think creatively, recognize the signs that a selected tool is not appropriate or effective and adapt or try something new. One strategy to achieve this could be to develop an individual interview schedule through the use of a short pilot interview (Guzman-Velez et al., 2014) or the creation of an individual communication repertoire where the individual’s communication methods, for example, eye gaze, movements and vocalizations can be observed and recorded (Downs & Collins, 2015).

## Summary and conclusions

The experiential opinion and expertise of the interviewees were employed to identify tools, methods and techniques to facilitate meaningful communication with people with moderate-to-severe dementia. Three overarching themes were identified, each reflecting key facilitating factors acting on both interlocutors to influence communication effectiveness. Building on the foundation of a genuinely person-centred approach to communication, personalized approaches using pictures, photographs and/or objects that are meaningful to the person can help to facilitate conversations. All identified elements of effective communication in the diagram should be considered as a whole when communicating with people who have moderate-to-severe dementia, particularly when aiming to elicit their preferences, views and opinions. In turn, this can assist with developing and evaluating new approaches and ensuring that services and support are relevant and responsive to individual needs.

## Supplemental Material

sj-pdf-1-dem-10.1177_14713012211069449 – Supplemental Material for Methods and approaches for enhancing communication with people with moderate-to-severe dementia that can facilitate their inclusion in research and service evaluation: Findings from the IDEAL programme Click here for additional data file. Supplemental Material, sj-pdf-1-dem-10.1177_14713012211069449 for Methods and approaches for enhancing communication with people with moderate-to-severe dementia that can facilitate their inclusion in research and service evaluation: Findings from the IDEAL programme by Rachel Collins, Anna Hunt, Catherine Quinn, Anthony Martyr, Claire Pentecost and Linda Clare in Dementia

sj-pdf-2-dem-10.1177_14713012211069449 – Supplemental Material for Methods and approaches for enhancing communication with people with moderate-to-severe dementia that can facilitate their inclusion in research and service evaluation: Findings from the IDEAL programme Click here for additional data file. Supplemental Material, sj-pdf-2-dem-10.1177_14713012211069449 for Methods and approaches for enhancing communication with people with moderate-to-severe dementia that can facilitate their inclusion in research and service evaluation: Findings from the IDEAL programme by Rachel Collins, Anna Hunt, Catherine Quinn, Anthony Martyr, Claire Pentecost and Linda Clare in Dementia
