# Supplementary material for: Methods and approaches for enhancing communication with people with moderate-to-severe dementia that can facilitate their inclusion in research and service evaluation: Findings from the IDEAL programme
Source: Dementia (London). 2022 Feb 13;21(4):1135–53. doi: 10.1177/14713012211069449 (PMC9109550; doi:10.1177/14713012211069449)
Supplement: sj-pdf-1-dem-10.1177_14713012211069449 – Supplemental Material for Methods and approaches for enhancing communication with people with moderate-to-severe dementia that can facilitate their inclusion in research and service evaluation: Findings from the IDEAL programme [file sj-pdf-1-dem-10.1177_14713012211069449.pdf]

Supplementary Table 1. Topic guide for semi-structured interviews with dementia professionals

**Introduction and general**
• Please can you say a few words to introduce yourself and describe your job role?<br>• How many years' experience do you have in this role?<br>• What experience do you have in communicating with people with dementia or other patient populations who have limited verbal ability?
**Existing Resources and Experience**
• How do you approach people with more progressed dementia and what do you do when you want to find out something about them or their experiences?<br>• Which of these techniques / tools works well and why?<br>• Which of these techniques / tools does not work well and why?<br>• What improvements would you suggest to existing tools to enable better communication with people with dementia?<br>• Have you ever used [Talking mats], [Pictures to Share books], [*other examples of tools*] or other existing resources, and what has been helpful or unhelpful about them in your experience?<br>• Are there any other resources / techniques you would like to discuss which you think work well / do not work well when improving communication in dementia?<br>• Do you know of resources / techniques from other areas of practice, such as learning difficulties or other patient populations, which work well as communication aids?<br>• Can you describe, from your experience, the ways people with dementia who are seldom heard in research, communicate or try to communicate?<br>• Is there anything not currently in use which you think would be helpful to improve communication with people with dementia?<br>• Do you think existing staff and researchers understand the ways people with advanced dementia communicate and what may help them better understand this?<br>• How do you think we could best capture the direct viewpoints, wishes and feelings of people with advanced dementia?
**Communication in different settings**
• Is there anything that needs to be considered when communicating with people with dementia in different settings - i.e. hospital/clinical setting, domestic home, care home, outdoors, public place?<br>• How may the communication needs differ for people with dementia in different settings and what do you think are the best resources to assist communication in

these respective settings?
**Communication with different audiences**
• Who do you think people with dementia communicate most easily with (family / care staff / strangers / other people with dementia), and what could we learn from this?<br><br>• What are the ways we might assist researchers and care staff to best understand how a person with dementia communicates?
**Personalization in communication**
• How could we adapt our approach to communication to ensure the individual needs and preferences for people with dementia are captured?<br><br>• Are there different techniques which may aid communication with different age groups in advanced dementia / different genders / nationalities / second language?
**Plus any extra questions that arise from the conversation**
