# Supplementary material for: Methods and approaches for enhancing communication with people with moderate-to-severe dementia that can facilitate their inclusion in research and service evaluation: Findings from the IDEAL programme
Source: Dementia (London). 2022 Feb 13;21(4):1135–53. doi: 10.1177/14713012211069449 (PMC9109550; doi:10.1177/14713012211069449)
Supplement: sj-pdf-2-dem-10.1177_14713012211069449 – Supplemental Material for Methods and approaches for enhancing communication with people with moderate-to-severe dementia that can facilitate their inclusion in research and service evaluation: Findings from the IDEAL programme [file sj-pdf-2-dem-10.1177_14713012211069449.pdf]

Supplementary Table 2. The final three main themes with the corresponding sub-themes and eighteen identified categories

Theme	Sub-theme	Initial categories
1.Awareness, knowledge, and experience	1a Cultural or Social contexts and challenges	Assumptions made of people with dementia
		Challenges for care staff
		Challenges for researchers
		Role of culture or background
	1b Transferable approaches	Comparison with other conditions
	1c Creative thinking and approaches	Creativity
2.Communication approach	2a Humanity and compassion	Humanistic approach
	2b Tangible techniques	Tools for supporting communication (creative arts, technology etc.)
	2c Interactional techniques	Non-verbal communication
		Role of storytelling, metaphors and reminiscence
		Communication Techniques or methods
3.Personalization	3a Environment and setting	Environment
		Influence of others
		Importance of giving time
	3b Getting to know each other	Observation
		Learning about the person
	3c Person-centered approach	Ethical considerations
		Benefits of communication for people with dementia
